# Perceptual impairment in face identification with poor sleep

**DOI:** 10.1098/rsos.160321

**Published:** 2016-10-05

**Authors:** Louise Beattie, Darragh Walsh, Jessica McLaren, Stephany M. Biello, David White

**Affiliations:** 1 School of Psychology, University of Glasgow, Glasgow, UK; 2 School of Psychology, UNSW Australia, Sydney, New South Wales, Australia

**Keywords:** face recognition, unfamiliar face matching, person identification, insomnia, sleep deprivation

## Abstract

Previous studies have shown impaired memory for faces following restricted sleep. However, it is not known whether lack of sleep impairs performance on face identification tasks that do not rely on recognition memory, despite these tasks being more prevalent in security and forensic professions—for example, in photo-ID checks at national borders. Here we tested whether poor sleep affects accuracy on a standard test of face-matching ability that does not place demands on memory: the Glasgow Face-Matching Task (GFMT). In Experiment 1, participants who reported sleep disturbance consistent with insomnia disorder show impaired accuracy on the GFMT when compared with participants reporting normal sleep behaviour. In Experiment 2, we then used a sleep diary method to compare GFMT accuracy in a control group to participants reporting poor sleep on three consecutive nights—and again found lower accuracy scores in the short sleep group. In both experiments, reduced face-matching accuracy in those with poorer sleep was not associated with lower confidence in their decisions, carrying implications for occupational settings where identification errors made with high confidence can have serious outcomes. These results suggest that sleep-related impairments in face memory reflect difficulties in perceptual encoding of identity, and point towards metacognitive impairment in face matching following poor sleep.

## 1. Introduction

The ability to verify identity by comparing images of faces is an important part of daily work in many security and forensic professions. These roles often entail long working hours combined with irregular shifts that can result in shortened sleep duration and insomnia, which can negatively impact cognitive performance [1,2]. However, previous research into the effects of sleep on face identification has measured effects of sleep restriction on memory consolidation; in recognition memory [3–5], perceptual adaptation [6] and perceptual learning tasks [7]. The impact of sleep restriction on face identification performance in commonplace tasks that do not involve memory, such as verifying photo-ID [8], has not been tested.

Consistent with the general importance of sleep for memory consolidation [9], a number of studies show that memory for faces is impaired after sleep restriction [4,5]. However, some studies examining effects of mild sleep deprivation did not find impairment in sleep-restricted participants (less than 7 h per night) [10] and the mechanisms underlying face memory deficit remain poorly understood [4]. Importantly, because this previous work measures memory for faces encountered prior to sleep, it is not clear whether sleep also impairs perceptual encoding of face identity, which would mediate any impact on recognition memory performance. Indeed, indirect neurophysiological evidence suggests that deficits in face memory following total sleep deprivation may arise from encoding difficulties [3], and there is some evidence for reduced perceptual sensitivity following sleep restriction [11]. It is, therefore, surprising that studies have not tested this question directly, by measuring accuracy in sleep-restricted participants on face identification tasks that do not rely on memory storage.

Matching identity of simultaneously presented face images may appear to be a straightforward task, but many studies have shown that performance is surprisingly poor when faces are unfamiliar. Even under optimal conditions, with images taken on the same day and in standard lighting, studies consistently find that people make a large proportion of errors [12,13]. When image capture conditions are not optimal, such as when matching images captured on CCTV, accuracy can approach chance levels [14]. This is particularly surprising because participants in these tasks were free to compare these images for as long as they required, and so the tasks place minimal demands on memory. Notably, professionals that perform the task in their daily work, such as police [14] and passport officers [8], do not perform any better—making the same proportion of errors as novice participants.

If sleep restriction impairs accuracy in face matching, this may contribute to poor performance in professional settings. Moreover, given large and stable individual differences in sleep behaviour and the ability to maintain vigilance after restricted sleep [15,16], sleep restriction may also contribute to the large inter-individual differences in accuracy that are typical in tests of face identification ability [8,13,17]. This inter-individual variance is theoretically informative, providing evidence to support the notion that face identification is supported by cognitive mechanisms specific to that purpose [17,18] and holding potential to reveal the functional architecture of the face processing system [19]. On a practical level, these differences suggest that staff selection can provide a practical solution to poor accuracy in professional settings [8], and so it is vitally important to identify underlying traits that propagate inter-individual variance in performance.

Here we test whether poor sleep impairs perceptual processing of faces by comparing performance in participants reporting poor sleep with control participants on the Glasgow Face-Matching Test (GFMT) [13]. Because unfamiliar face matching is an important component of daily work across a number of security and forensic professions, we were motivated to examine face-matching ability in non-clinical populations with sleep disruption, to reflect levels of sleep disruption common among shift workers. In Experiment 1, we tested GFMT performance in individuals reporting sleep behaviour consistent with insomnia, which is a common sleep disorder in the general population [20]. In Experiment 2, we then clarified whether impairments in face matching are associated with short sleep more generally. In both experiments, we asked participants to rate their confidence in face-matching decisions—both for practical reasons and to clarify conflicting evidence regarding the effects of sleep on ratings of confidence [21,22].

## 2. Experiment 1

### 2.1. Material and methods

#### 2.1.1. Participants

Participants were 102 volunteers who visited the Glasgow Sleep Research Group stall at the Glasgow Science Centre (74 female) with ages ranging from 16 to 74 (*M* = 37.9 years, s.d. = 14.0). Insomnia was identified if participants scored below threshold on the *Sleep Condition Indicator* (SCI, see procedure below), resulting in a normal sleep group (*n* = 77, 55 females, mean age = 37.1 years, s.d. = 14.4) and an insomnia group (*n* = 25, 19 females; mean age = 40.3 years, s.d. = 12.9). All participants completed the GFMT before completing three sleep questionnaires.

#### 2.1.2. Glasgow Face-Matching Task

**Figure 1.**
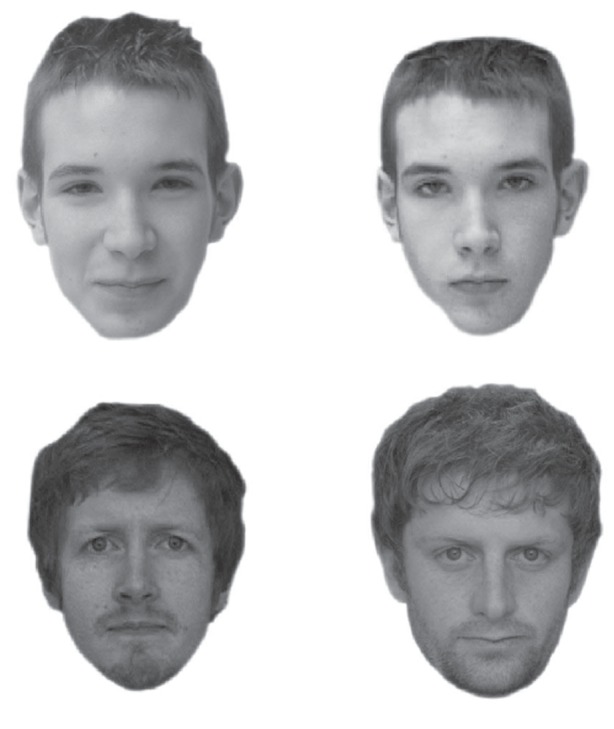
Example image pairs from the Glasgow Face-Matching Test, reproduced from a previous publication [13]. The top row shows a same identity pair and the bottom row shows a different identity pair.

The GFMT [13] consists of 40 sequentially presented image pairs. Example test items of each type are shown in figure 1. Half of the image pairs show two images of the same person (match trials), and half show two different people (mismatch trials). For match trials, images are captured with two different cameras, but on the same day, under similar lighting conditions and in the same neutral pose. For mismatch trials, images are of two similar looking people. Face pairs are shown in a random order and for each pair participants must decide if the images are of the same person or two different people. The task is self-paced, meaning that participants are able to freely inspect each image repeatedly and for as long as they require before reaching a decision. After each same/different response, participants rated their confidence in their decision on a scale from 1 to 100.

#### 2.1.3. Sleep measures

After completing the GFMT, participants answered three sleep questionnaires. The SCI [23] is a measure of insomnia disorder over the past month. This questionnaire comprises eight questions that score from 0 to 4, where lower scores indicate poorer sleep, and overall scores of 16 or below are diagnostic of insomnia disorder. The *Pittsburgh Sleep Quality Index* (PSQI) [24] quantified participants' quality of sleep during the preceding month. This questionnaire comprises seven component scores, such as sleep latency and sleep disturbances, and overall scores range from 0 to 21, with higher scores indicating poorer sleep. The *Epworth Sleepiness Scale* (ESS) [25] assessed participants' general level of daytime sleepiness. This questionnaire asks participants about their likelihood of dozing in eight everyday scenarios, with higher scores indicating greater sleepiness.

### 2.2. Results

Table 1 shows summary sleep measure scores for groups in Experiment 1. Group differences in SCI scores confirmed there were significantly more symptoms of insomnia in the insomnia group (*t*_100_ = −13.41, *p* < 0.001, Cohen's *d* = 3.08). There were also significant group differences in PSQI scores (*t*_100_ = 9.82, *p* < 0.001, Cohen's *d* = 2.12), suggesting more sleep disruption in the insomnia group over the previous month. Group differences in ESS scores were non-significant (*t*_100_ = 0.45, *p* = 0.66, Cohen's *d* = 0.10) consistent with previous reports of hyperarousal in insomnia disorder [26].

**Table 1.** Summary sleep measure scores for groups in Experiment 1.

	group					
	normal sleepers			insomnia		
	mean	median	s.d.	mean	median	s.d.
SCI	25.7	26.0	4.53	11.7	14.0	4.55
PSQI	3.92	4.00	2.28	9.44	9.00	2.89
ESS	7.18	6.00	4.13	7.64	7.00	5.31

**Figure 2.**
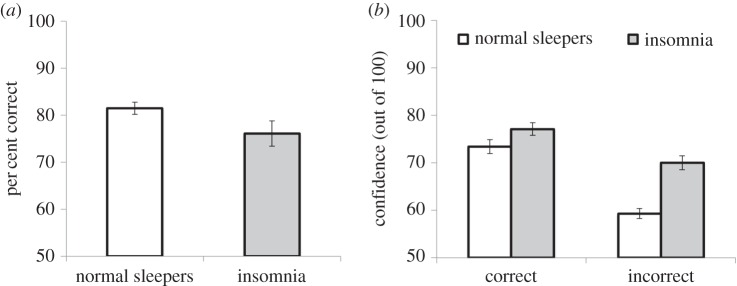
GFMT accuracy (*a*) and confidence (*b*) for groups in Experiment 1. Error bars represent standard error.

Accuracy and confidence on the GFMT for insomnia and normal sleeper groups are summarized in figure 2. There was a marginally significant difference between groups in overall accuracy, with poorer face-matching accuracy in the insomnia group (*t*_100_ = 1.96, *p* = 0.05, Cohen's *d* = 0.43). This difference appeared to be driven by a significantly reduced rate of correct rejections (i.e. more false alarms) in the insomnia group (*t*_100_ = 2.35, *p* = 0.02, Cohen's *d* = 0.48). Differences between hit rates in the two groups were non-significant (*t*_100_ = 0.41, *p* = 0.68, Cohen's *d* = 0.09). Interestingly, differences in accuracy were not accompanied by corresponding differences in confidence. Indeed, when incorrect, the insomnia group were *more* confident in their decisions than control participants (*t*_97_ = 3.03, *p* = 0.003, Cohen's *d* = 0.72). There were no significant group differences in confidence when correct (*t*_100_ = 1.03, *p* = 0.31, Cohen's *d* = 0.28). Analysis also confirmed non-significant differences in reaction times for all response types (all *p*s > 0.36).

## 3. Experiment 2

Results of Experiment 1 suggest that people with higher rates of insomnia-related symptoms are more likely to make errors in unfamiliar face matching. Given that these symptoms are prevalent in the general population, it is somewhat concerning that insomnia participants were *more* confident when making face-matching errors, showing that poorer accuracy on this task was not associated with awareness of the impairment. In Experiment 2, we aimed to clarify whether short sleep duration more generally is associated with poorer face-matching performance, by measuring sleep duration using a sleep diary over three nights prior to completing the GFMT.

### 3.1. Material and methods

#### 3.1.1. Participants

Sixty-two participants volunteered to participate. Twelve were excluded because they did not meet *a priori* inclusion criteria, either because they had: (i) irregular sleep patterns in the three days prior to testing, and so did not meet criteria for either the short sleep or control group, or (ii) were control participants that showed signs of a primary sleep disorder (see details of exclusion criteria below). This resulted in a final sample of 25 control subjects (15 females; mean age = 23.8 years, s.d. = 4.07) and 25 participants with short sleep (13 females; mean age = 24.7 years, s.d. = 5.76).

#### 3.1.2. Sleep measures

We used a sleep diary to calculate participants' total sleep duration per night and sleep efficiency measures. Sleep efficiency was measured as total sleep duration divided by the total time spent in bed over the three nights. Sleep duration was used to determine group allocation with short sleep participants defined as sleeping for less than or equal to 6.5 h on each night for the three nights prior to performing the GFMT. Control subjects had slept at least 7 h in each of the three nights preceding testing. Eight participants who failed to meet either criterion were excluded from the study.

The ESS and PSQI, described in Experiment 1, were used to quantify the levels of sleep disruption in the short sleep group, in additional to two further sleep measures. The Karolinska Sleepiness Scale (KSS) [1] measured participants' level of subjective sleepiness during testing on a scale from 1 (very alert) to 9 (very sleepy). The Insomnia Severity Index (ISI) [27] measured symptoms of insomnia, and was also used to identify participants in the control group who indicated probable signs of insomnia (score of 15 or more) and/or perceived themselves as having insomnia. Two control participants were excluded based on ISI scores. The sleep disorders algorithm [28] was then used to identify participants in the control group who exhibited signs of a sleep disorder. This algorithm screens for narcolepsy, sleep breathing disorder, periodic limb movements/ restless legs syndrome, circadian rhythm sleep disorder and parasomnia via the use of an initial ‘lead’ question. A further two participants were removed from the control group on the basis of the sleep disorder algorithm.

#### 3.1.3. Procedure

Each participant was given a sleep diary three days prior to testing. They were instructed to fill out the details of their sleep for three consecutive nights. Following the third night, participants returned to the laboratory where they completed the GFMT [13]. As in Experiment 1, we used the short version of this test and confidence ratings were collected after each response. After completing the GFMT, participants then completed the sleep questionnaires.

### 3.2. Results

Sleep measures are summarized in table 2. We observed significant group differences in PSQI (*t*_48_ = −5.19, *p* < 0.001, Cohen's *d* = 1.53), ISI (*t*_48_ = −5.09, *p* < 0.001, Cohen's *d* = 1.43) and KSS scores (*t*_48_ = −5.50, *p* < 0.001, Cohen's *d* = 1.56), but no significant group differences in the ESS (*t*_48_ = −1.48, *p* = 0.15, Cohen's *d* = 0.418), confirming state sleepiness and poorer sleep quality in the short sleep group. There were significant group differences in sleep efficiency (*t*_48_ = 2.79, *p* = 0.008, Cohen's *d* = 0.78) and sleep duration (*t*_48_ = 13.36, *p* < 0.001, Cohen's *d* = 3.77) scores over the three days prior to testing. Thus, the short sleep group had experienced poorer sleep quality over the previous month, had greater insomnia severity in the week prior to testing and had poorer sleep efficiency in the three days before testing. This group also reported greater sleepiness at testing, with the expected differences in sleep duration for the three nights prior to testing.

**Table 2.** Sleep measures for groups in Experiment 2.

	group					
	control			short sleep		
	mean	median	s.d.	mean	median	s.d.
PSQI	4.40	4.00	1.85	8.24	8.00	3.21
ISI	4.64	4.00	3.95	11.1	12.0	5.00
KSS	3.12	3.00	1.64	5.80	7.00	1.80
ESS	8.28	8.00	3.67	9.80	10.0	3.59
sleep duration (h)	8.01	7.92	0.59	5.16	5.33	0.89
sleep efficiency (%)	91.5	94.9	6.95	83.8	82.9	12.1

**Figure 3.**
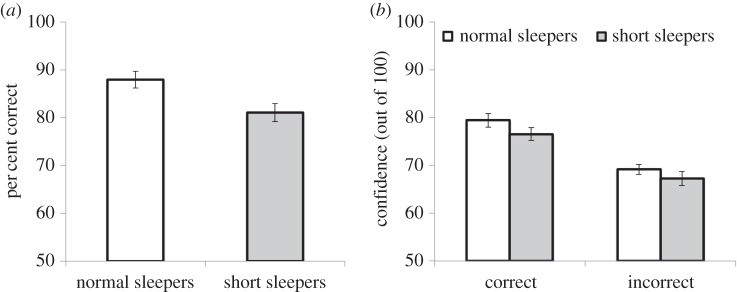
GFMT accuracy (*a*) and confidence (*b*) for groups in Experiment 2. Error bars represent standard error.

Performance data for the GFMT are summarized in figure 3. Overall, the short sleep group made more errors on face matching than normal sleepers (*t*_48_ = 2.61, *p* = 0.012, Cohen's *d* = 0.74). This result was largely driven by match trials (i.e. reduced hit rates in short sleepers), with the short sleep group making a greater proportion of miss responses relative to control participants (*t*_48_ = 2.49, *p* = 0.016, Cohen's *d* = 0.77). The short sleep group tended to also make more errors in non-matching pairs (i.e. false alarms) although this difference was non-significant (*t*_48_ = 1.49, *p* = 0.143, Cohen's *d* = 0.42).

Although less accurate than control participants, the short sleep group were not less confident. For both correct (*t*_48_ = 1.54, *p* = 0.13, Cohen's *d* = 0.30) and incorrect responses (*t*_45_ = 0.522, *p* = 0.604, Cohen's *d* = 0.04) ratings of confidence did not differ significantly between short sleep group and controls. Differences in response latency between groups were non-significant for all response types (*p* > 0.55).

## 4. General discussion

In both experiments, participants with impaired sleep made more errors in a standardized test of face-matching ability. The GFMT involves matching identity of images presented simultaneously, confirming for the first time that deficits in face identification accuracy associated with restricted sleep are not limited to tasks that rely on recognition memory. This result has important implications for understanding the mechanisms underlying impairments in face recognition, entailing that deficits in perceptual encoding processes underpin impairment in face recognition, and may also modulate processing impairments in facial emotion caused by poor sleep [29–31].

Individual performance on the GFMT ranged from 40% correct to 100% correct, consistent with previous studies showing large individual differences in face identification accuracy [8,12]. Research examining correlates of individual differences in face identification accuracy have expanded rapidly over recent years, driven by strong evidence for a stable genetic basis for these differences [17,18]. Our results show that individual differences in sleep behaviour are very likely to contribute to these individual differences. However, we have not yet established a causal relationship between sleep and face matching, raising the possibility that higher levels of depressed mood and anxiety in insomnia [32], for example, may mediate the association between poor sleep and reduced face-matching accuracy [33]. It will be important in future work to establish a causal effect between sleep restriction and unfamiliar face matching by experimentally restricting sleep. This will enable studies to examine how individual differences in sleep behaviour [15], in combination with resilience to sleep restriction [16] affect face-matching performance. This is likely to have important implications for ascertaining the causes of individual differences in face processing ability, and also for the design of selection processes to enlist staff for critical security roles [7,34,35].

Our results may also have a bearing on the nature of visual perception impairments in sleep-restricted participants more generally. There is strong evidence for a general role of sleep in memory consolidation [9], and perceptual learning of objects [7]. However, very few studies have examined perceptual discrimination in tasks that do not involve memory or learning, and visual discrimination of simple stimuli appears to be unaffected by sleep loss [35]. This raises the question of whether impairments after restricted sleep are related to the complexity of the visual task. One possibility, given established sleep-impairments for guided visual attention [16], is that performance on visual tasks that are demanding of attentional mechanisms is impaired after short sleep [36]. This possibility is consistent with recent evidence that selective attention contributes to high levels of accuracy in unfamiliar face-matching tasks [37].

Despite impaired accuracy in the GFMT, poor sleepers in both experiments were no less confident in their responses. Indeed, the insomnia group in Experiment 1 were *more* confident in errors than normal sleepers. This overconfidence suggests that awareness of matching accuracy may also be reduced after sleep disruption. Although contrary to studies reporting null effects of sleep deprivation on confidence-accuracy calibration in a simple line judgement perceptual task [22], this result may reflect a more general impairment in executive function and risk assessment after sleep restriction [38]. The practical implications of this result are concerning. Overconfidence entails that errors in identification are not only more common after interrupted sleep, but are also more likely to remain undetected. Thus in security and forensic settings, where face matching underpins many important identity verification tasks [8,34,37], sleep disruption is likely to exacerbate a general overconfidence in unfamiliar face-matching tasks [39]. Incorrect judgements made with high confidence can have serious consequences; for example, causing known insurgents to be undetected in CCTV footage or enabling criminals to obtain fraudulent identity documents [34]. Given the prevalence of shift work in security professions, efforts should be made to mitigate this risk.

In summary, this study implicates a role for perceptual processes in sleep-related impairments of face identification. This is the first evidence that these impairments are not limited to recognition memory tasks that rely on memory consolidation processes. Future experimental work should aim to delineate relative contributions of attention, perception and working memory to this impairment. In addition to improving understanding of the effects of sleep in critical security tasks, this may also help to elucidate the cognitive hierarchy underpinning people's ability to identify faces.

## Supplementary Material

S1: Performance and sleep measure data for Experiment 1

## Supplementary Material

S2: Performance and sleep measure data for Experiment 2

## Data accessibility

All data created during this research are openly available from Dryad data repository: http://dx.doi.org/10.5061/dryad.r620r [40].
